# Supplementary figures and images for: Breed-Specific Variations in Vertebral Right Heart Index (VRHi): Implications for Detection of True and False Right Heart Enlargement (RHE) in Dogs
Source: Vet Sci. 2025 Mar 24;12(4):300. doi: 10.3390/vetsci12040300 (PMC12031339; doi:10.3390/vetsci12040300)

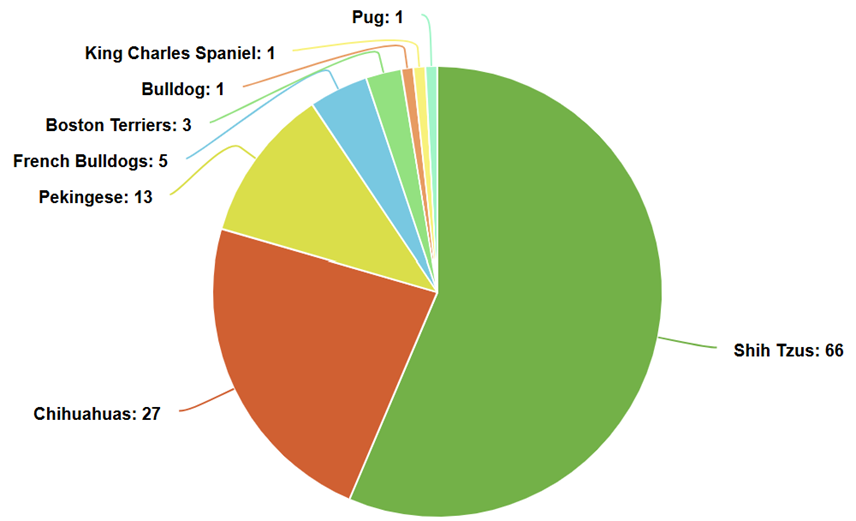

Supplement: Supplementary file 1 [file vetsci-12-00300-s001.zip › supplementary materials_ S2pie chart of brachycephalic group.png]

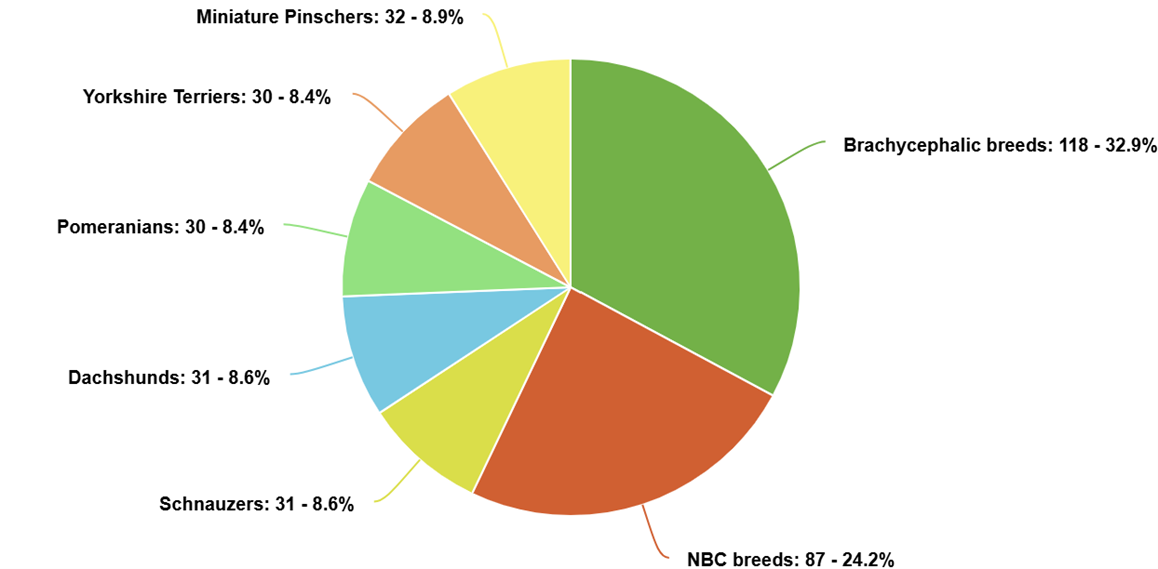

Supplement: Supplementary file 1 [file vetsci-12-00300-s001.zip › supplementary materials_S1pie chart of entire group.png]

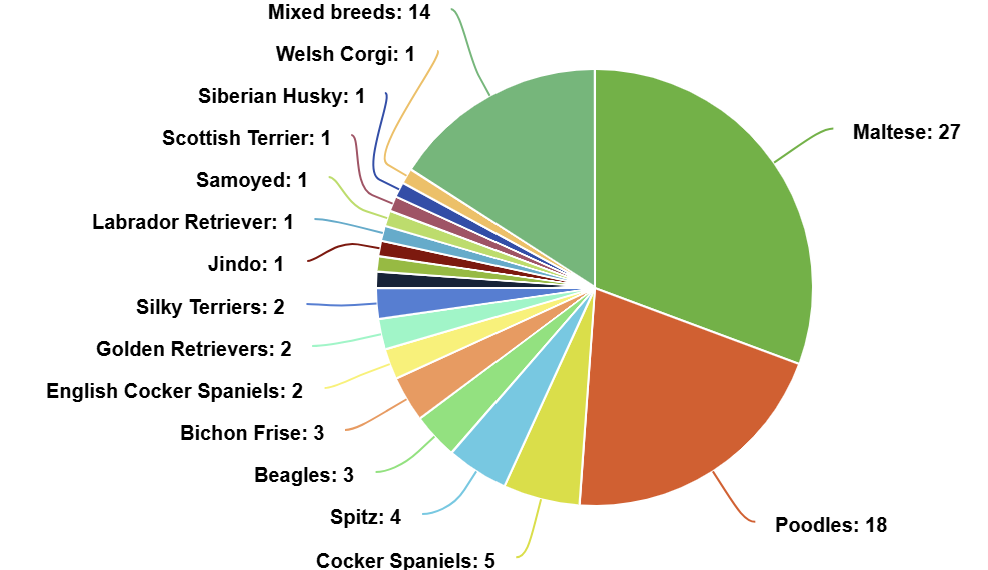

Supplement: Supplementary file 1 [file vetsci-12-00300-s001.zip › supplementary materials_S3pie chart of NBC group.png]
